# Re-irradiation of spinal column metastases by IMRT: impact of setup errors on the dose distribution

**DOI:** 10.1186/1748-717X-8-269

**Published:** 2013-11-16

**Authors:** Christian Gröger, Matthias G Hautmann, Rainer Loeschel, Natalia Repp, Oliver Kölbl, Barbara Dobler

**Affiliations:** 1 Department of Radiotherapy, University Medical Center, Regensburg, Germany; 2 Department of Computer Science and Mathematics, University of Applied Sciences, Regensburg, Germany

**Keywords:** CBCT, Setup error, IGRT, IMRT, Spinal metastasis

## Abstract

**Background:**

This study investigates the impact of an automated image guided patient setup correction on the dose distribution for ten patients with in-field IMRT re-irradiation of vertebral metastases.

**Methods:**

10 patients with spinal column metastases who had previously been treated with 3D-conformal radiotherapy (3D-CRT) were simulated to have an in-field recurrence. IMRT plans were generated for treatment of the vertebrae sparing the spinal cord. The dose distributions were compared for a patient setup based on skin marks only and a Cone Beam CT (CBCT) based setup with translational and rotational couch corrections using an automatic robotic image guided couch top (Elekta - HexaPOD™ IGuide^®^ - system). The biological equivalent dose (BED) was calculated to evaluate and rank the effects of the automatic setup correction for the dose distribution of CTV and spinal cord.

**Results:**

The mean absolute value (± standard deviation) over all patients and fractions of the translational error is 6.1 mm (±4 mm) and 2.7° (±1.1 mm) for the rotational error. The dose coverage of the 95% isodose for the CTV is considerable decreased for the uncorrected table setup. This is associated with an increasing of the spinal cord dose above the tolerance dose.

**Conclusions:**

An automatic image guided table correction ensures the delivery of accurate dose distribution and reduces the risk of radiation induced myelopathy.

## Background

The spinal column belongs to the most common sites for cancer cells to metastasize [1]. These metastases are often associated with spinal cord compression resulting in significant pain and neurological deficits [2]. Radiotherapy has shown to be a proven method to relieve pain and prevent complications [3-5]. However, a considerable percentage of patients develop a metastatic recurrence within the initially irradiated region. For these patients in-field re-irradiation can be an option [6,7]. Though the risk of myelopathy has to be minimized by assuring that the summed spinal cord dose does not exceed a certain tolerance value [8-11]. Modern radiation techniques like cyberknife treatment [12], stereotactic body radiotherapy (SBRT) [13,14], intensity modulated radiotherapy (IMRT) [15-18] or volumetric modulated arc therapy (VMAT) [19,20] reduce the dose to the spinal cord and allow an effective treatment of the vertebra bone metastasis at the same time. This is accomplished by creating a steep dose gradient between tumor tissue and organ at risk, so that in case of spinal column metastases the radiation sensible spinal cord can be protected during the treatment planning process. The application of such highly locally resolved treatment techniques tighten the problem of a locally very precise treatment delivery to avoid severe damage to the patient. A precise dose delivery including a very accurate patient setup is essential for the positive outcome of the treatment avoiding tumor underdosing and overdosing of critical structures. Image guided (IG) treatment techniques like cone beam computed tomography (CBCT) in combination with an automated patient setup correction system is an established technic to correct and verify the patient setup [18,21,22].

This study investigates the impact of an automated patient setup correction by the Elekta XVI-IGuide^®^-HexaPOD™ system on the dose distribution for ten patients simulating an in-field re-irradiation of vertebral metastases using IG-IMRT techniques. The dose distribution of patient setup using only skin marks is compared with the dose distribution after an automated setup correction.

## Methods

### Equipment

Treatment planning was performed with Oncentra^®^ External Beam v4.1. Patients were irradiated with a Synergy^®^ S (Elekta Ltd, Crawley, UK) linear accelerator with Beam Modulator™ head (21 cm × 16 cm max. field, 4 mm leaf width at isocenter). The Synergy^®^ S Platform is equipped with a XVI Cone Beam CT (CBCT) with a flat panel detector. For precise patient setup the patient couch (Precise Table, movable in 3 translation directions) is additionally upgraded with a robotic HexaPOD™ Couch Top (Medical Intelligence, Schwabmünchen, Germany), which allows translations and rotations in six independent degrees of freedom. This system uses an infrared camera system (IGuide^®^, Elekta) for automated patient setup correction.

### Patients

10 patients with spinal column metastases who had previously been treated with 3D-CRT (1st treatment course) were selected from our treatment database for this retrospective study. The irradiated region includes 1–5 thoracic vertebrae (including spinal cord). For this study it was assumed that these patients have an in-field recurrence and the whole vertebra region (including the spinal canal) was pretreated with a dose of 10 × 3 Gy in the first course. For the second course the CTV consisted of one to five whole vertebra including the vertebral body, the vertebral arch, the transverse processes and the spinous process. The spinal canal was excluded from the CTV. The PTV was defined as CTV + 3 mm in each direction excluding the spinal canal from this expansion. The biological equivalent dose BED was calculated according to the formula BED = n ∙ d ∙ (1 + d/(α/β)) [8], where n is the number of fractions, d is the dose per fraction and α and β are the linear and quadratic coefficients of linear-quadratic cell killing probability. According to the literature the α/β value for the spinal cord tissue was assumed to be 2 Gy [8]. Therefore the BED of the first radiation course of 10 × 3 Gy is BED_1_ = 75 Gy_2_. Based on the risk score model of Nieder et al. [8,9], the risk of radio myelopathy appears small after a summed BED (BED_tot_) < 120 Gy_2_ when the interval is not shorter than 6 months and the dose of each course is < 98 Gy_2_. Due to the fact that the BED is additive the BED of the spinal cord tissue of the second treatment course should not exceed 45 Gy_2_ (BED_2_) [4,23]. The dose prescription for the second course was 6 × 4 Gy (BED_2_ = 72 Gy_2_, BED_tot_ = 147 Gy_2_). In order to minimize the risk of radio myelophaty only a dose of 6 × 3 Gy (75% of the prescribed dose, BED_2_ (spinal cord) = 45 Gy_2_) is allowed at the spinal cord for the 2nd course (the calculation is described in [15]).

### Treatment planning

IMRT plans were generated for each patient allowing coverage of the target region while sparing the spinal cord. The following objectives for planning were applied:

– PTV: The 95% isodose covering the outer PTV (a dose deficit in the spinal cord direction is allowed to establish an dose gradient and spare the spinal cord)

– Spinal Cord: D_0.1ccm_ < 18 Gy, i.e. the doses exceeding 18 Gy is delivered to a volume smaller than 0.1 ccm to avoid radio myelopathy. 18 Gy equates to 75% of the prescribed dose.

– The region of the dose gradient from PTV to spinal cord should be minimized.

CT-slice thickness and dose grid were set to 2 mm. All plans were optimized using collapsed cone dose calculation algorithm with 9 isotropically distributed beam directions. Max. 80 segments and “step and shoot” technique were used. The prescribed dose is normalized to the average of a structure which is equivalent to the PTV minus 3 mm margin around the spinal cord. The isocenter was set to the middle of the spinal cord.

### Patient setup errors

Before the first treatment the correct position of the isocenter was checked at the simulator by taking sagittal and coronal x-ray images. Skin marks were placed to localize the isocenter. During the 1st treatment course CBCTs in treatment position were acquired for each patient: Patients are positioned according to the skin marks and a CBCT was acquired and registered with the planning CT by automatic matching the outlines of the bones in both CT data sets. The setup error values were automatically calculated from the software and transferred to the HexaPOD™ system for an automatic table shift. New skin markers for the isocenter were placed if a table correction was performed in the same direction at two successive days. The first six setup error values of the 1st treatment course were taken as hypothetical setup errors for this study. A comparison was performed between the dose distribution calculated for the uncorrected CT data set and for the corrected CT dataset which is identical to the planning CT. The uncorrected CT data set was created by shifting and rotating the planning CT dataset with an in-house developed software according to the setup error values above for each fraction. Afterwards, the original treatment plan is calculated on this new CT. The dose distribution of each fraction is summed up to a total dose, which represents the dose distribution for the patient without performing a XVI based setup correction. The translational setup error values are defined as follows: x axis (left-right), y axis (cranio-caudal) and z axis (anterior-posterior); and for the rotational errors: α (pitch, i.e. rotation around x-axis), β (roll, i.e. rotation around y-axis) and γ (yaw, i.e. rotation around z-axis).

### Dose parameters

The following parameters are evaluated from the DVH to specify the quality of dose distributions before and after setup correction with the HexaPOD™ system. Percentage dose values refer to the prescribed dose D_AVG_(PTV) = 24 Gy.

CTV and PTV:

– Dose coverage V_95%_: Percentage of volume receiving 95% of the prescribed dose

– Average dose D_AVG_

– Minimum dose D_99%_: Percentage of prescribed dose which covers 99% of the volume

– Maximum dose D_01%_: Percentage of prescribed dose which covers 1% of the volume

– Homogeneity index: H = (D_99%_-D_01%_)/D_AVG_

Spinal cord (SC):

– Maximum dose D_0.1ccm_: Percentage of prescribed dose which covers 0.1 ccm of the volume

– Minimum dose D_99%_: Percentage of prescribed dose which covers 99% of the volume

– Average dose D_AVG_(SC)

– V_75%_(SC) : absolute volume receiving more than 75% of the prescribed dose

## Results

### Setup error

Absolute values (± standard deviation) of the translational and rotational setup errors are given in Table 1. The mean translational errors (± standard deviation) over all patients and fractions were 6.1 mm (±4 mm) and 2,7° (±1.1 mm) for the rotational error.

**Table 1:** Risk evaluation

	**Absolute error 3D vector**		**Max dose D**_**01ccm**_		**BED (Gy**_**2**_**)**		**Risk factor**
	**Translation (mm)**	**Rotation (°)**	**%**	**Gy**	**Max dose**	**Total**	
**Patient 1**	10.2	3.3	86.7	20.8	56.8	131.8	2
**Patient 2**	5.6	3.3	85.3	20.5	55.4	130.4	2
**Patient 3**	4.2	2.8	78.6	18.9	48.5	123.5	1
**Patient 4**	10.0	3.7	94.8	22.8	65.9	140.9	3
**Patient 5**	6.1	3.1	88.4	21.2	58.7	133.7	2
**Patient 6**	5.4	3.0	94.2	22.6	65.2	140.2	3
**Patient 7**	5.9	2.1	82.9	19.9	52.9	127.9	1
**Patient 8**	2.8	2.1	78.1	18.7	48.0	123.0	1
**Patient 9**	4.3	2.0	79.9	19.2	49.8	124.8	1
**Patient 10**	6.2	1.7	82.2	19.7	52.2	127.2	1
**mean value**	6.1	2.7					

Absolute error, maximum dose, BED and risk factor for each patient in case HexaPOD™ correction is not performed.

### Dose distribution

**Figure 1:**
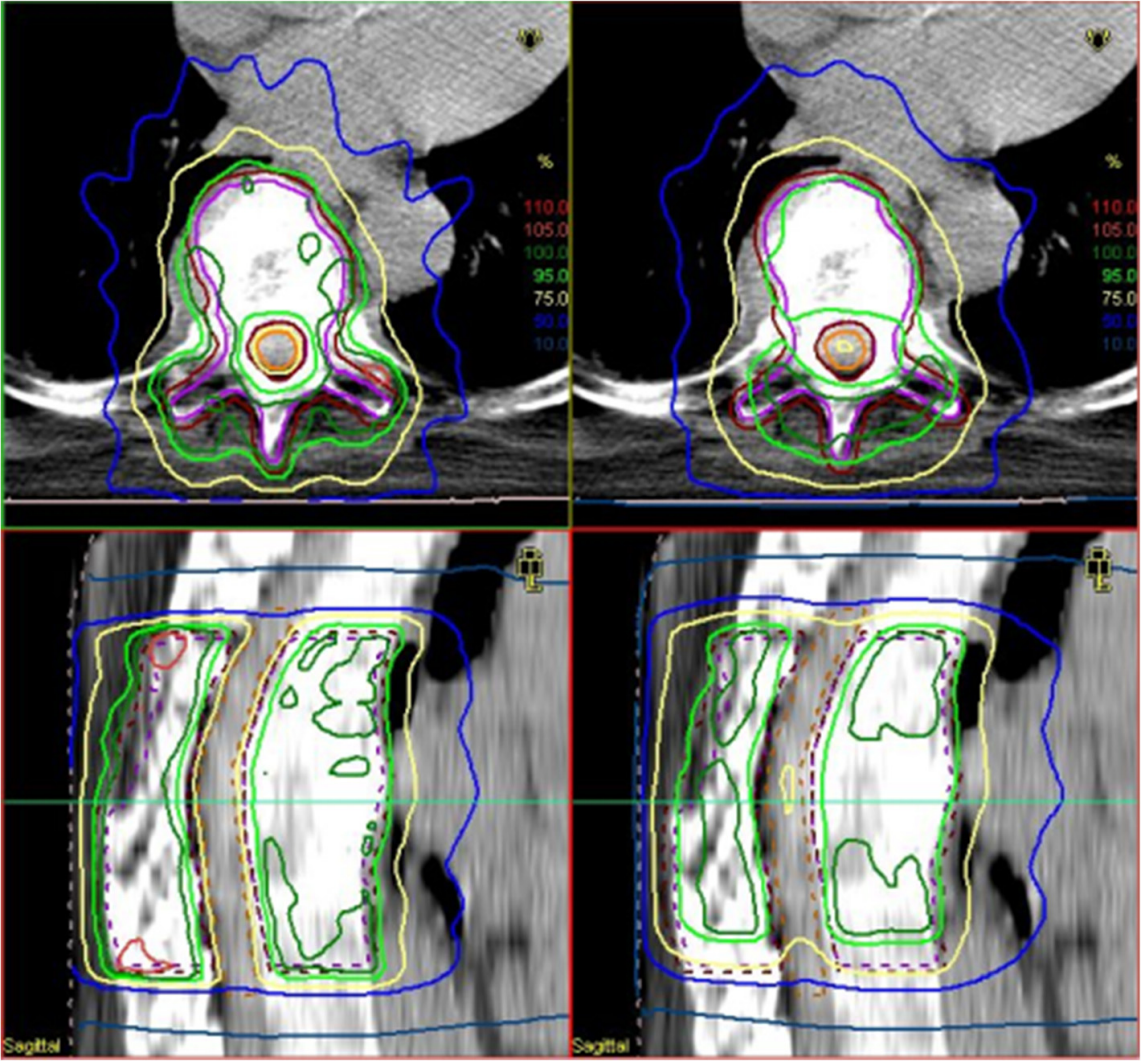
**Dose distributions for patient 1.** Dose distribution (isocentric transversal slice: top and isocentric sagittal slice: bottom) for patient 1 before (right) an after (left) setup correction. Displayed are isodose lines for 50% (12 Gy - blue), 75% (18 Gy - yellow), 95% (22,8 Gy - light green), 100% (24 Gy - dark green) and 105% (25,2 Gy - light red); CTV is marked in purple, PTV in brown red and spinal cord in orange.

Table 2 gives a summary of the dose values for the evaluated regions of interest CTV, PTV and spinal cord. The most obvious differences in the dose distribution of CTV and PTV can be seen at the parameter V_95%_. The mean value is considerably decreased from 86.6% to 81.6% for CTV and from 89.9% to 79.4% for PTV, respectively if the table position is not corrected with the HexaPOD™ system. The highest difference in the CTV is observed for patient 1: 89.6% (corrected) – 71.3% (uncorrected). Figure 1 shows the dose distribution for patient 1 before (right) and after (left) setup table correction. The lowest difference is given for patient 6: 84.3% (corrected) – 83.6% (uncorrected).

**Table 2:** Evaluated dose parameters

		**Corrected**		**Uncorrected**		**t-test**
		**Mean**	**SD**	**Mean**	**SD**	**p value**
**CTV**	**V**_**95%**_	86.6	1.9	81.6	3.8	0.0036
	**D**_**AVG**_	98.9	0.3	98.4	1.2	0.3284
	**D**_**99%**_	79.6	2	78	1.5	0.0860
	**D**_**01%**_	104.8	0.7	104.3	1.4	0.3413
	**H**	0.26	0.03	0.27	0.02	0.3383
**PTV**	**V**_**95%**_	89.8	1.4	79.4	6.3	0.0007
	**D**_**AVG**_	99.6	0.2	97.7	1.2	0.0010
	**D**_**99%**_	80.4	1.7	76.4	3.5	0.0091
	**D**_**01%**_	105.3	0.7	103.8	1.4	0.0148
	**H**	0.25	0.02	0.28	0.03	0.0329
**SC**	**(D**_**0.1ccm**_**)%**	75.1	0.5	85.1	5.7	0.0005
	**(D**_**0.1ccm**_**)Gy**	18.0	0.1	20.4	1.4	0.0005
	**D**_**99%**_	37.2	14.6	38.3	13.6	0.8731
	**D**_**AVG**_	63.3	1.7	68.9	3.5	0.0008
	**(V**_**75%**_**)ccm**	0.1	0.0	3.7	3.6	0.0160

Comparison of evaluated dose values with and without HexaPOD™ setup correction. A p-value of less than 0.05 is assumed to be significant.

**Figure 2:**
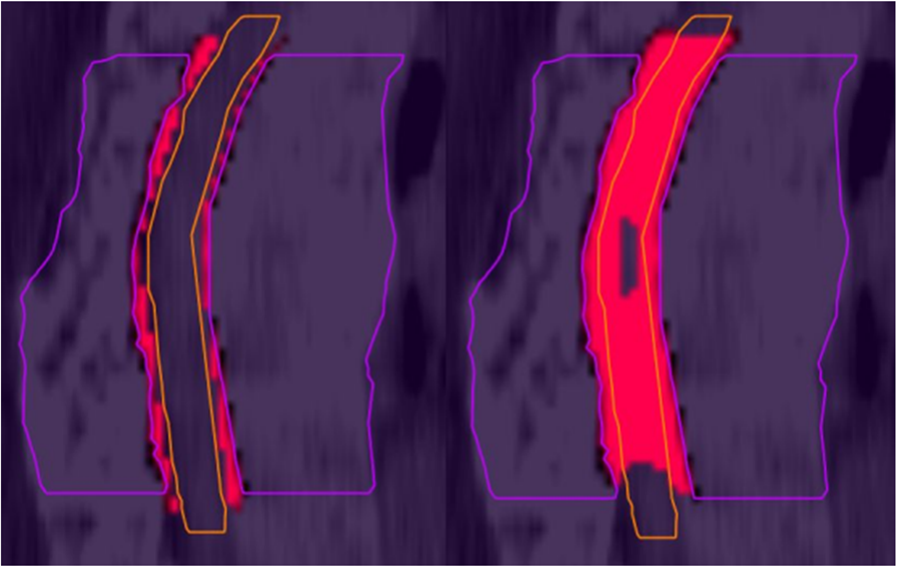
**Hot spots inside the spinal canal.** Sagittal icocentric slice for patient 1 with (left) and without (right) CBCT table setup correction. Hotspots (dose over the allowed tolerance dose of 18 Gy) inside the spinal canal are displayed in red. CTV is marked in purple and spinal cord in orange.

The evaluated dose parameters for the spinal cord are listed in Table 2. The mean maximal dose D_0.1ccm_ is significantly increased from 75.1% of the prescribed dose (18.0 Gy) to 85.1% (20.4 Gy). For instance patient 4 obtains a maximum of 94.8% of the prescribed dose (22.8 Gy) in case the patient setup is performed by skin marks only and is not corrected with the HexaPOD™ system. This is accompanied with an increase of the spinal cord volume which receives more than 75% of the prescribed dose. The mean value V_75%_(SC) grows from 0.1 ccm to 3.7 ccm. Figure 2 shows the hot spot areas inside the spinal canal for patient 1 before (right) and after (left) setup table correction. The converted maximum dose in BED is given in Table 1 for each patient.

## Discussion

The low radiation tolerance of spinal cord limits the possibility of irradiation of in-field recurrence of spinal cord metastases. Nieder et al. [8,9] evaluated published clinical data of 78 patients who received an in-field re-irradiation of spinal vertebra metastases. Due to these data they established a risk score to estimate the probability of myelopathy after in field re-irradiation based on the applied total biologically effective dose (BED). The risk score ranges from 0 (total BED ≤ 120: very low risk) to 9 (total BED > 200: very high risk). Our patients have already received an exposure dose of BED = 75 Gy_2_ during the first treatment course. According to Nieder et al. a dose of BED = 120 Gy_2_ at the spinal cord is considered as limit. In this study we use IMRT for the second treatment course to create a steep dose gradient between tumor region and spinal cord and maintain the limit.Patient setup errors during the treatment are responsible for deviation between planned and applied dose distribution. This study shows the impact of an automated patient setup correction by the Elekta XVI-IGuide^®^-HexaPOD™ system to the dose distribution for ten patients simulating an in-field re-irradiation of vertebral metastases using IMRT techniques. The dose distribution of patient setup using skin marks only is compared with the dose distribution after an automated setup correction. The most sensitive parameter in our case is the dose at the spinal cord. The total BED for a patient setup performed only by skin marks is given in Table 1: All patients obtain a higher dose than BED = 120 Gy_2_ at the spinal cord which is the maximum dose what was allowed during planning. Therefore a risk factor of 0 could not be accomplished without CBCT setup correction. 2 patients received even a dose of BED > 140 Gy_2_ and a risk factor of 3 with an increased risk of myelopathy. Apart from the maximum dose at the spinal cord the absolute volume which receives an increased dose is also regarded to be important. Therefore the spinal cord volume V_75%_ was also evaluated. The mean value increases from 0.1 ccm to 3.7 ccm. Patient 1 shows even an increase of the volume to 12.5 ccm (see Figure 2). Due to the requirement of a steep dose gradient a dose coverage of 100% to PTV or CTV could not be achieved in the initial planning. Our analysis shows that patient setup errors have an additional effect on dose coverage of the PTV and CTV. The dose coverage V_95%_ is considerably decreased from 89.9% to 79.4% (PTV) and 86.6% to 81.6% (CTV). Further studies have to be performed to judge the clinical relevance of this additional loss in dose coverage.

## Conclusions

This study shows that the method of patient setup substantially affects the geometric accuracy of the dose delivery. A patient setup using skin marks only exposes the patients to risks of radiation induced myelopathy. On the other hand the application of an automatic IG patient setup like the Elekta - HexaPOD™ IGuide^®^ - system ensures the delivery of accurate dose distribution and reduces the risk of radiation induced myelopathy in case of in-field re-irradiation of vertebral metastases.

## Competing interests

This work was partly supported by Elekta.

## Authors' contributions

CG collected the data, performed data analysis and evaluation and drafted the manuscript. MGH participated in the collection and assessment of patient data and in manuscript revision. BD and OK participated in the design and coordination of the study and in manuscript revision. NR and RL developed the software for the conversion of the CT datasets according the patient setup errors. All authors read and approved the final manuscript.
